# The role of exercise-and high fat diet-induced bone marrow extracellular vesicles in stress hematopoiesis

**DOI:** 10.3389/fphys.2022.1054463

**Published:** 2022-11-23

**Authors:** James J. Vanhie, Wooseok Kim, Lisa Ek Orloff, Matthew Ngu, Nicolas Collao, Michael De Lisio

**Affiliations:** ^1^ School of Human Kinetics, Faculty of Health Sciences, Ottawa, ON, Canada; ^2^ Department of Cellular and Molecular Medicine, Faculty of Medicine, University of Ottawa, Ottawa, ON, Canada

**Keywords:** physical activity, obesity, microRNA, myelopoiesis, adipogenesis

## Abstract

Exercise and obesity regulate hematopoiesis, in part through alterations in cellular and soluble components of the bone marrow niche. Extracellular vesicles (EVs) are components of the bone marrow niche that regulate hematopoiesis; however, the role of exercise training or obesity induced EVs in regulating hematopoiesis remains unknown. To address this gap, donor EVs were isolated from control diet-fed, sedentary mice (CON-SED), control diet-fed exercise trained mice (CON-EX), high fat diet-fed, sedentary mice (HFD-SED), and high fat diet-fed, exercise trained mice (HFD-EX) and injected into recipient mice undergoing stress hematopoiesis. Hematopoietic and niche cell populations were quantified, and EV miRNA cargo was evaluated. EV content did not differ between the four groups. Mice receiving HFD-EX EVs had fewer hematopoietic stem cells (HSCs) (*p* < 0.01), long-term HSC (*p* < 0.05), multipotent progenitors (*p* < 0.01), common myeloid progenitors (*p*<0.01), common lymphoid progenitors (*p* < 0.01), and granulocyte-macrophage progenitors (*p* < 0.05), compared to mice receiving HFD-SED EVs. Similarly, mice receiving EX EVs had fewer osteoprogenitor cells compared to SED (*p* < 0.05) but enhanced mesenchymal stromal cell (MSC) osteogenic differentiation in vitro (*p* < 0.05) compared to SED EVs. HFD EVs enhanced mesenchymal stromal cell (MSC) adipogenesis in vitro (*p* < 0.01) compared to CON EVs. HFD-EX EVs had lower microRNA-193 and microRNA-331-5p content, microRNAs implicated in inhibiting osteogenesis and leukemic cell expansion respectively, compared to HFD-SED EVs. The results identify alterations in EV cargo as a novel mechanism by which exercise training alters stress hematopoiesis and the bone marrow niche.

## Introduction

Hematopoiesis, the process of blood cell formation from hematopoietic stem cells (HSCs), occurs in the bone marrow and is regulated by complex interactions between HSCs and their niche. Cellular components of the HSC niche include mesenchymal stem/stromal cells (MSCs), endothelial cells, osteoblasts, adipocytes, neurons, and as well as HSC progeny at various stages of differentiation. MSCs are particularly relevant due to their role in regulating hematopoiesis through their secretome and forming other cellular constituents of the HSC niche through their differentiation. Bone marrow cells communicate *via* paracrine mechanisms that have not been completely described. A greater appreciation for the role in lifestyle factors in regulating the HSC niche and hematopoiesis has recently developed. Preclinical models of diet-induced obesity results in an accelerated accumulation of bone marrow adipose tissue *via* promoting the adipogenic differentiation of MSCs, that is, related to the overproduction of inflammatory myeloid cells from HSCs (Emmons et al., 2019). This aberrant myelopoiesis is believed to contribute to systemic inflammation in obesity. Conversely, data from our lab and others have shown that participation in increased levels of physical activity and exercise training reduces marrow adipose tissue due to increased osteogenic MSC differentiation, reverses aberrant myelopoiesis, and decreases HSC turnover under obesogenic conditions (Styner et al., 2014; Emmons et al., 2019). The paracrine mechanisms responsible for the effects of obesity and physical activity/exercise training on hematopoiesis and the bone marrow niche have not been completely described.

Systemically, obesity is characterized by chronic low-grade inflammation, that is, accompanied by elevated levels of active immune cells in peripheral tissues that secrete pro-inflammatory cytokines (Benova and Tencerova, 2020). In bone marrow, inflammation induces HSC proliferation that promotes pre-emptive HSC pool exhaustion and skews HSC differentiation along the myeloid lineage to form common myeloid progenitor cells (CMPs), granulocytes, and monocytes, thus further exacerbating systemic inflammation (Trottier et al., 2012; Singer et al., 2014). This myeloid cell skewing from HSCs is due, in part, to increased marrow adipose tissue content formation from MSCs (Ambrosi et al., 2017). Styner and colleagues previously linked diet-induced obesity to marrow adipose tissue content formation in mice through high-fat diet (HFD) feeding, finding that exercise was capable of mitigating adipocyte production (Styner et al., 2014). Furthermore, other reports have shown that bone marrow adipocytes are capable of promoting HSC proliferation and differentiation (Poloni et al., 2013; Zhang et al., 2019). Recent work from our lab and others has shown that exercise influences hematopoiesis through alterations to the HSC niche. For example, we have previously shown that acute exercise stimulates HSC mobilization from the bone marrow and alters MSC gene expression (Emmons et al., 2016). Other work from our lab investigating exercise training surrounding a single exposure to sublethal whole-body irradiation found lower adipose tissue accumulation and differentially expressed soluble factors in the marrow of exercise trained mice compared to sedentary groups (Frodermann et al., 2019). Furthermore, Frodermann and colleagues identified leptin signaling from adipocytes and C-X-C motif chemokine ligand 12 (CXCL12) from MSCs as key mediators of HSC expansion (Frodermann et al., 2019). The current literature suggests that paracrine signaling between bone marrow cells regulate obesity- and exercise-induced alterations in hematopoiesis (Poloni et al., 2013; Emmons et al., 2016, 2019; Zhang et al., 2019).

EVs are membrane-bound particles that are secreted by all cells and act as intercellular mediators of communication (Yáñez-Mó et al., 2015; Doyle and Wang, 2019). EVs transfer miRNA and protein to alter recipient cell activity and fate (Ratajczak et al., 2006; Valadi et al., 2007; Skog et al., 2008). Szatmári and colleagues found that injecting EVs from irradiated mice into non-irradiated mice reduced HSC content comparable to direct radiation treatment (Szatmári et al., 2017). Conversely, Wen and colleagues showed non-irradiated MSC-derived EV injections into irradiated mice improves circulating white blood cell concentration recovery, thereby suggesting a role for EVs in promoting stress hematopoiesis (Wen et al., 2016). These findings demonstrate that EVs influence HSC activity and fate. To date, there has not been a full characterization of the paracrine factors regulating hematopoiesis with exercise and HFD. However, previous reports have shown exercise impacts EV production. In skeletal muscle, miR-486 is downregulated following a bout of acute exercise (Aoi et al., 2013); miR-486 has also been linked to erythrocyte formation, thereby indicating a systemic role for EVs in affecting hematopoiesis (Shi et al., 2017). Other reports have shown exercise training increases total circulating EV and circulating endothelial cell-derived EV concentrations (Bei et al., 2017; Ma et al., 2018).

Although prior studies have examined the role of EVs in regulating stress hematopoiesis (Wen et al., 2016; Szatmári et al., 2017), no study has investigated the alterations to bone marrow-derived EV cargo by exercise or HFD, or their impact on HSC and HSC niche cell content. Therefore, the purpose of the current study was to examine the effects of EVs derived from exercise trained and sedentary mice given a HFD or control (CON) diet on stress hematopoiesis in naïve mice. We hypothesized that EVs from mice fed a HFD would increase HSC and adipocyte progenitor cell concentrations in the bone marrow, and that EVs from exercise-trained mice would attenuate these effects.

## Materials and methods

### Experimental design

**FIGURE 1:**
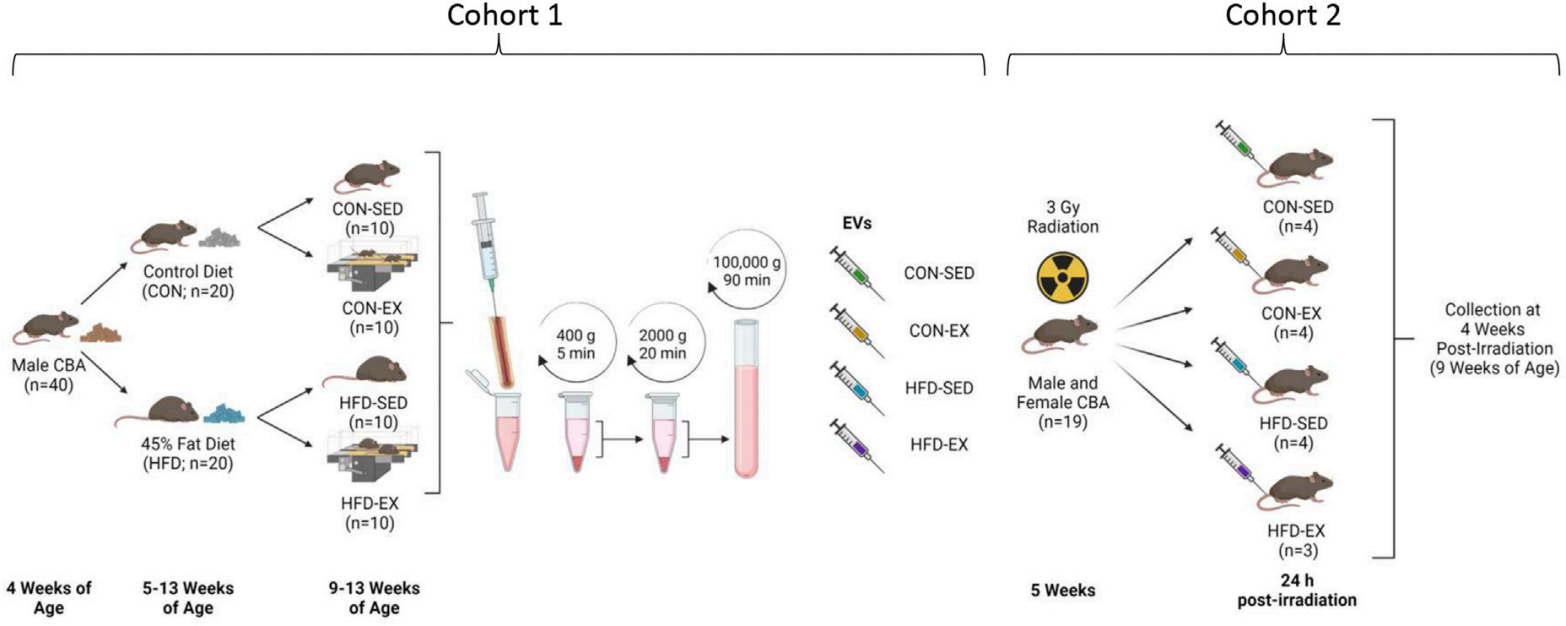
Study outline for EV isolation and injection. Male CBA mice (*n* = 40) were placed on high-fat (HFD) or control (CON) diets for 8 weeks with the latter 4 weeks including exercise (EX) or sedentary (SED) conditions. Bone marrow was extracted, and extracellular vesicles (EVs) isolated by differential centrifugation. Collected EVs were combined by group and injected into new male and female CBA mice by tail vein 24 h following ionising radiation injury. Bone marrow samples were collected at 4 weeks post-irradiation. Created with BioRender.com.

All protocols were approved by the University of Ottawa Animal Care Committee in accordance with the *Animals for Research Act* and by the Canadian Council on Animal Care. Mice were maintained on a 12:12 h light-dark schedule with food and water provided *ad libitum.* Cohort 1 was used to derive EVs. For the first cohort, male CBA (n = 40; Jackson Laboratories, United States) mice aged 5 weeks were randomly selected for 8 weeks of 45% fat (HFD; D12451, Research Diets, NJ, United States) or control (CON; D10012M, Research Diets, NJ, United States) diet feeding (Figure 1). After 4 weeks on their respective diets, half of the mice in each condition were randomly selected for exercise training (EX) while the rest remained sedentary (SED). Cohort 2 mice were EV recipients. For Cohort 2, male and female CBA mice (n = 3-4 per group) were irradiated with 3 Gy of whole-body gamma radiation using a X-Rad 320 biological irradiator (Precision X-Ray, Madison, Connecticut, United States) to induce stress hematopoiesis. At 24-h post-irradiation, Cohort 2 mice were injected with bone marrow-derived EVs isolated from non-irradiated Cohort 1 mice. Cohort 2 mice remained sedentary and were fed standard chow (Teklad 2018 Rodent Diet, Envigo, Indianapolis, IN, United States) *ad libitum* throughout the study. Four weeks after EV injection, bone marrow was collected for cell content analysis from Cohort 2 mice.

### Endurance test and exercise training

Mice in Cohort 1 underwent an endurance test using a Exer 3/6 treadmill (Columbus Instruments, Columbus, OH, United States) angled upwards at 5°, as described previously, to evaluate training status (Baker et al., 2011; De Lisio et al., 2011, 2013; De Lisio and Parise, 2012; Emmons et al., 2019; Farber et al., 2021). All mice were acclimated for 10 min at 8 m min^−1^ the week prior to the endurance test. Cohort 1 mice ran at 10 m min^−1^ and increased speed once every 2 minutes by 1 m min^−1^ until the mice were resistant to running or were unable to keep their hind limbs on the treadmill for one full stage despite gentle encouragement. The mice were encouraged to run using a soft-bristle paint brush; electric shock was not used for any test. Following the endurance test, Cohort 1 mice underwent an exercise training program, as described previously (Baker et al., 2011; Emmons et al., 2019; Farber et al., 2021). The exercise training program involved three exercise sessions per week for 40–60 min, depending on the training week. The exercise protocol consisted of a warm-up period at 8 m min^−1^ for 10 min for the first 3 weeks (10 m min^−1^ for week 4) followed by an 8–10 m min^−1^ training speed for 25− (week 1), 35− (week 2), or 45− (weeks 3, 4) min and a cooldown period at 8 m min^−1^ for 5 min. Sedentary mice were placed in sham treadmills on top of the running treadmill to account for any stress associated with the treadmill vibrations, noise, and handling.

### Extracellular vesicle characterization, injection, and miRNA quantification

EVs were isolated from the bone marrow supernatant *via* differential ultracentrifugation as described previously, with minor modifications (Lobb et al., 2015). Briefly, bone marrow was extracted from Cohort 1 mice femurs and tibias using a 25-gauge needle and 1 ml of phosphate-buffered saline (PBS), followed by a 400 g centrifugation cycle at 4°C for 5 min. The supernatant was stored at −80°C for later analysis and injection. After thawing, the supernatant was centrifuged at 2000 g at 4°C for 20 min to remove any remaining cell fragments and apoptotic bodies. The supernatant was removed and ultracentrifuged thereafter at 100,000 g at 4°C for 90 min using an Optima MAX-micro-Ultracentrifuge (Beckman Coulter, Brea, CA, United States). EV pellets were resuspended with 110 µL sterile PBS, of which 10 µL was used for concentration and size distribution analysis at 1:100 dilution using the ZetaView PMX 110 Multiple Parameter Particle Tracking Analyzer (Particle Metrix, Meerbusch, Germany). Relative to *in vitro* isolation, bone marrow offers a small pool of EVs. Across all groups, we found that our bone marrow EV yield averaged 420 million particles per sample. Based on our quantification we were able to combine grouped samples to increase the EV injection counts to 650 million per mouse in Cohort 2, with some remaining samples available for follow-up miRNA content and *in vitro* C3H 10T1/2 MSC adipogenic and osteogenic analysis. Following EV quantification, EVs were combined by exercise and dietary condition and injected via tail vein. Mice were weighed prior to injection with sample volumes standardised across each mouse to 8 μL/g body weight. EV isolation was confirmed by Western blot using 30 µg of protein derived from six combined bone marrow EV samples and MSC lysate. Antibodies included anti-TSG101 (T5701, Sigma-Aldrich), anti-Alix (SAB4200476, Sigma-Aldrich), anti-Flotillin-2 (#3436S, Cell Signaling Technology) diluted 1:1000 in 5% bovine serum albumin-diluted tris-buffered saline with 1% Tween 20 (TBST). Horseradish peroxidase-linked secondary antibody (7074S, Cell Signaling Technology) was diluted 1:10,000 in 5% milk-TBST. The membranes were incubated for 5 min using SuperSignal West Pico PLUS enhanced chemiluminescent substrate (ThermoFisher Scientific, MA, United States) and visualized using a ChemiDoc Imaging System (Bio-Rad, CA, United States).

EV miRNA cargo from individual mice in Cohort 1 (*n* = 3 per group) was isolated using the Qiagen miRNEasy Micro kit (Hilden, Germany) according to the manufacturer’s instructions. Extracted miRNA sample integrity and concentrations were measured using a Nanodrop 2000 (Thermo Fisher Scientific, Waltham, MA, United States), with concentrations ranging from 47.7 to 166.6 ng/μL. Using 100 ng of isolated miRNA, specific miRNA content expression was identified using the Nanostring mouse v1.5 miRNA kit as per the manufacturer’s instructions (Nanostring, Seattle, WA, United States). The prepared cartridges were read using a Nanostring nCounter station. One CON-SED and two CON-EX samples did not pass quality control measures on Nanostring’s proprietary nSolver 4.0 software and were excluded from statistical analysis. The HFD-SED and HFD-EX samples were analyzed using the ROSALIND analysis program (Seattle, WA, United States). Briefly, the nCounter-derived RCC files for the HFD-SED and HFD-EX conditions (*n* = 3 per condition) were uploaded to the ROSALIND website. The samples were normalized by positive control and codeset normalization with fold-changes and significance calculated by the Student’s t-test. Absolute-fold changes of ± 1.25 or greater (the default value by ROSALIND) and a *p*-value lower than 0.05 were considered significant. miRNAs that were differentially expressed underwent further investigation for potential gene targets using the miRWalk miRNA target mining feature (http://mirwalk.umm.uni-heidelberg.de/). All differentially expressed miRNAs were inserted to the search list and subsequent gene set enrichment analysis was selected for KEGG and GO:BP enrichment pathways. The top five results for the Kyoto Encyclopedia of Genes and Genomes (KEGG) and Gene Ontology: Biological Processes (GO:BP) enrichment pathways were recorded for literature investigation.

### Bone marrow collection and cell quantification

Mice were euthanized at 4 weeks post-EV injection by CO_2_ asphyxiation followed by cervical dislocation. Femurs and tibiae were isolated for bone marrow extraction. Phosphate-buffered saline (PBS) was flushed at least 6 times through the bone marrow cavity to maximize bone marrow tissue collection. The cell suspension was centrifuged at 400 g at 4°C for 5 min and the EV-rich supernatant was frozen at −80°C immediately. The remaining cell pellet was gently resuspended with 5% fetal bovine serum (FBS) with PBS and stored on ice until further processing. To extract any remaining cells, the bones were crushed once with a pestle and mortar, cut into tiny fragments, and chemically digested for 45 min with 0.2% type II collagenase diluted in high glucose DMEM. The cells were triturated every 15 min to promote chemical digestion. Bone marrow content from flushing and bone digestion were combined, filtered through a 70 µm filter, and centrifuged at 400 g at 4°C for 5 min. The pellets were collected and resuspended in 5% FBS for flow cytometry staining.

Cohort 2 bone marrow bone marrow cells were quantified using an Attune NxT flow cytometer (Thermo Fisher Scientific), as described previously (De Lisio and Parise, 2012; Emmons et al., 2016, 2019). All cell identification strategies are summarized in Table 1, and all antibodies, fluorophores, and associated catalog numbers are summarized in Table 2. Live cells were used for the Zombie Yellow viability dye compensation. The flow cytometry gating was based on unstained and single-stained bone marrow cells (Supplemental Figures S1–S3).

**TABLE 1:** Flow cytometry gating strategies for HSPC and niche cell populations.

Cell population	Gating strategy
Hematopoietic Stem Cells (HSCs)	Lineage^−^, Sca-1^+^, c-Kit^+^ (LSK)
Long-Term HSCs	LSK, CD48^−^, CD150^+^
Short-Term HSCs	LSK, CD48^−^, CD150^-^
Multipotent Progenitors	LSK, CD48^+^, CD150^-^
Common Myeloid Progenitors	LSK, CD16/32^int^, CD34^int^
Common Lymphoid Progenitors	Lineage^−^, Sca-1^int^, c-Kit^-^
Granulocyte-Monocyte Progenitors	LSK, CD16/32^hi^, CD34^+^
Megakaryocyte-Erythroid Progenitors	LSK, CD16/32^-^, CD34^−^
Mesenchymal Stromal Cells	Ter119^-^, CD45^−^, CD31^−^, CD51^+^, CD140α^+^
Endothelial Cells	Ter119^-^, CD45^−^, CD31^−^, CD51^−^
Adipocyte Progenitors	Ter119^-^, Sca-1^+^, CD45^−^, CD31^−^, CD51^−^
Osteoblasts	Ter119^-^, Sca-1^-^, CD45^−^, CD31^−^, CD51^+^
Osteoprogenitors	Ter119^-^, Sca-1^+^, CD45^−^, CD31^−^, CD51^−^

**TABLE 2:** Antibodies used for flow cytometry.

Antibody	Conjugate	Dilution	Product number
CD16/32	BV711	1:200	101337
CD31	BV510	1:200	563089
CD34	Pe-Cyanine5	1:200	119312
CD45	PE-Cyanine7	1:200	552848
CD48	BV510	1:200	563536
CD51	BV421	1:200	740062
Sca1 (Ly6A/E)	PE	1:200	553336
cKit (CD117)	PE-Cyanine7	1:200	558163
CD140a	PE	1:200	562776
CD150	BV421	1:200	562811
Lineage Panel (5)	Biotin	1:200 (5:200 total)	559971
Streptavidin	FITC	1:800	554060
Viability	Zombie Yellow	1:300	423104

### C3H 10T1/2 proliferation and differentiation assays

C3H 10T1/2 cells are a model of bone marrow stromal cells that are capable of induced osteogenic and adipogenic differentiation (Shea et al., 2003; Huang et al., 2009). To observe the effects of HFD- and EX-induced EVs on MSC proliferation and differentiation directly, C3H 10T1/2 cells were plated on 96-well plates (1250 cells per well) with 100 µL of growth media (10% FBS, 1% penicillin-streptomycin, in high-glucose Dulbecco’s Modified Eagle Medium; DMEM) and incubated at 37°C for 72 h to reach 90% confluence. Cells were then given either adipogenic or osteogenic differentiation media. For the initial plating and each concurrent media change, cells were supplemented with 5.1 million EVs. This value represented approximately twice the EV concentration injected into the mice and was selected to directly measure the effects of HFD- and EX-EVs on MSC differentiation and/or proliferation. Adipocyte differentiation was induced using the Mesencult adipogenic differentiation kit according to the manufacturer’s instructions (Stemcell Technologies, Vancouver, BC, Canada). Two days after adding differentiation media, cells were supplemented with 1 μg/ml of insulin to induce adipocyte cell maturation for another 3 days. After 5 days of differentiation media supplementation, cells were fixed in 10% formalin on a rocker for 30 min at room temperature, washed twice with 100 µL of distilled water, and stained with 100 µL of 0.5% Oil Red O dye on a rocker for 30 min at room temperature while protected from light. Unbound dye was washed away by washing the wells 3 times with distilled water. The remaining bound dye was removed by adding 200 µL of isopropanol and was incubated on a rocker at room temperature for 10 min while protected from light. 100 µL of the isopropanol-Oil Red O dye solution was transferred to a new 96-well plate and measured by spectrophotometry at 492 nm. Osteogenesis was induced using the Mesencult osteogenic differentiation kit according to the manufacturer’s instructions (Stemcell Technologies). Osteogenic differentiation media was replaced every 3 days for 14 days. After 14 days of differentiation, cells were fixed in 10% formalin on a rocker for 30 min at room temperature, washed twice with 100 µL of distilled water, and stained with 100 µL of 0.2% Alizarin Red S dye on a rocker for 20 min at room temperature while protected from light. Alizarin Red S preparation and extraction was conducted as outlined by Gregory and colleagues using 10% of the listed volumes to account for the 96-well plate volume capacity (Gregory et al., 2004). Absorbance was read on a spectrophotometer at 405 nm.

The effects of HFD- and EX-EVs on C3H 10T1/2 cell proliferation were measured using the Alexa Fluor 488 Click-iT EdU cell proliferation kit according to the manufacturer’s instructions (C10337, Thermo Fisher Scientific). Briefly, 1250 cells were added to each well in a 96-well plate with 100 µL of growth media, as described above, and were incubated for 2 h at 37°C to allow cell adhesion to the plate. After 2 h, 50 µL of the cell culture media was removed and replaced with 50 µL of 20 µM EdU labeling solution (for a final concentration of 10 µM) containing 5.1 million EVs per well. Cells were fixed with 100 µL 4% formaldehyde at 6-, 12-, 24-, and 48-h post-media change, washed twice with 100 µL of 3% BSA in PBS, and permeabilized with 100 µL 0.5% Triton X-100 in PBS. To detect EdU-incorporated cells 50 µL of the Click-iT Plus reaction cocktail (85.76% 1X Click-iT reaction buffer, 4% Copper protectant, 0.24% Alexa Fluor picolyl azide, 10% 1X Click-iT EdU buffer additive) was added to each well and incubated for 30 min at room temperature while protected from light. Wells were then washed with 100 µL 3% BSA in PBS and again with 100 µL PBS. To stain all DNA, 1X Hoeschst 33342 (5 μg/ml) was added to each well and incubated for 30 min at room temperature while protected from light. The wells were then washed twice with 100 µL PBS. Plates were imaged and analyzed with a ZEISS CellDiscoverer7 microscope.

### Statistical analysis

Flow cytometry and C3H 10T1/2 *in vitro* proliferation and differentiation data were analyzed using a two-factor (diet and exercise) ANOVA followed by a Sidak post-hoc analysis for interaction effects. The two-factor ANOVAs were carried out using GraphPad Prism 9 (GraphPad Software). A Student’s t-test was conducted by ROSALIND software to compare HFD-SED and HFD-EX miRNA data. All data are presented as mean ± SEM with *p* < 0.05 being considered statistically significant. Investigators were blinded to all files and experimental groups for all analyses.

## Results

### Exercise increases extracellular vesicle concentration at specific sizes

**FIGURE 2:**
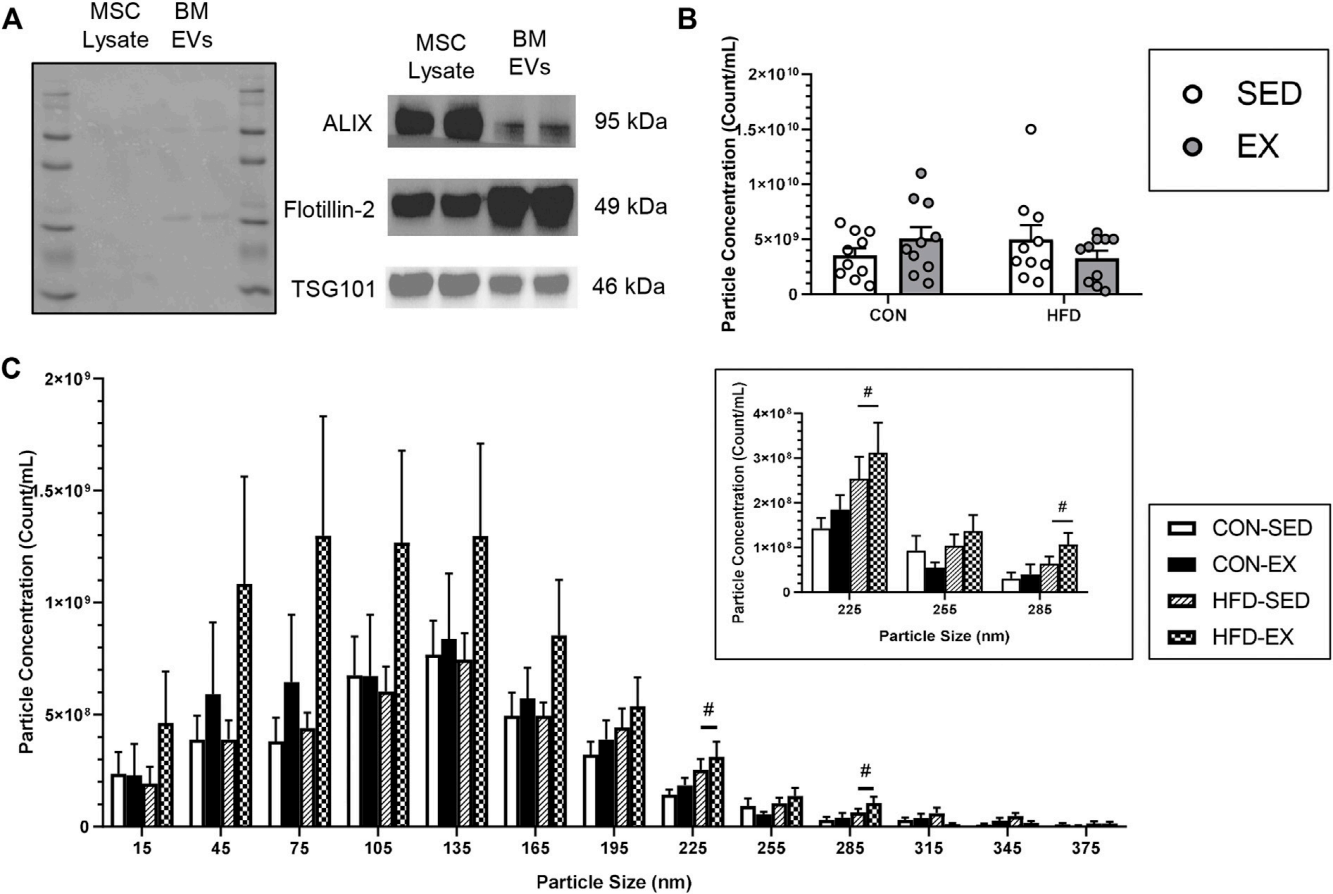
Characterization of extracellular vesicle (EVs) isolated from bone marrow supernatant of high-fat (HFD) or control (CON) diet-fed mice placed on exercise (EX) or sedentary (SED) conditions. **(A)** EV presence confirmation by mesenchymal stromal cell (MSC) lysate positive control measures of ALIX, Flotillin-2, and TSG101 proteins, **(B)** Total extracted particle concentration measured by nanoparticle tracking analysis (*n* = 10 per group), and **(C)** EV size distribution of injected EVs. ^#^ *p* < 0.05, high-fat diet difference *versus* control diet (*n* = 10 per group).

EV isolation was confirmed through Western blot analysis using the positive markers; ALIX, Flotillin-2, and TSG 101, according to recommended guidelines (Willms et al., 2016; Théry et al., 2018), with MSC lysate included as a positive control (Figure 2A). NTA revealed no difference in average particle concentration between any groups (*n* = 10 per condition) (Figure 2B). Most EV particles were smaller than 200 nm in size, an expected observation as a large proportion of extracellular vesicles are small EVs that are typically 30–200 nm in size (Maumus et al., 2020). However, EV concentration was higher in CON-EX *vs.* CON-SED at 225 ± 15 nm (*p* < 0.05), and there was a main effect of exercise having higher EV concentrations at 315 ± 15 and 345 ± 15 nm (*p* < 0.05) (Figure 2C).

### Extracellular vesicles from exercise training mice reverse HSC expansion and myeloid progenitor cell skewing with HFD during stress hematopoiesis

**FIGURE 3:**
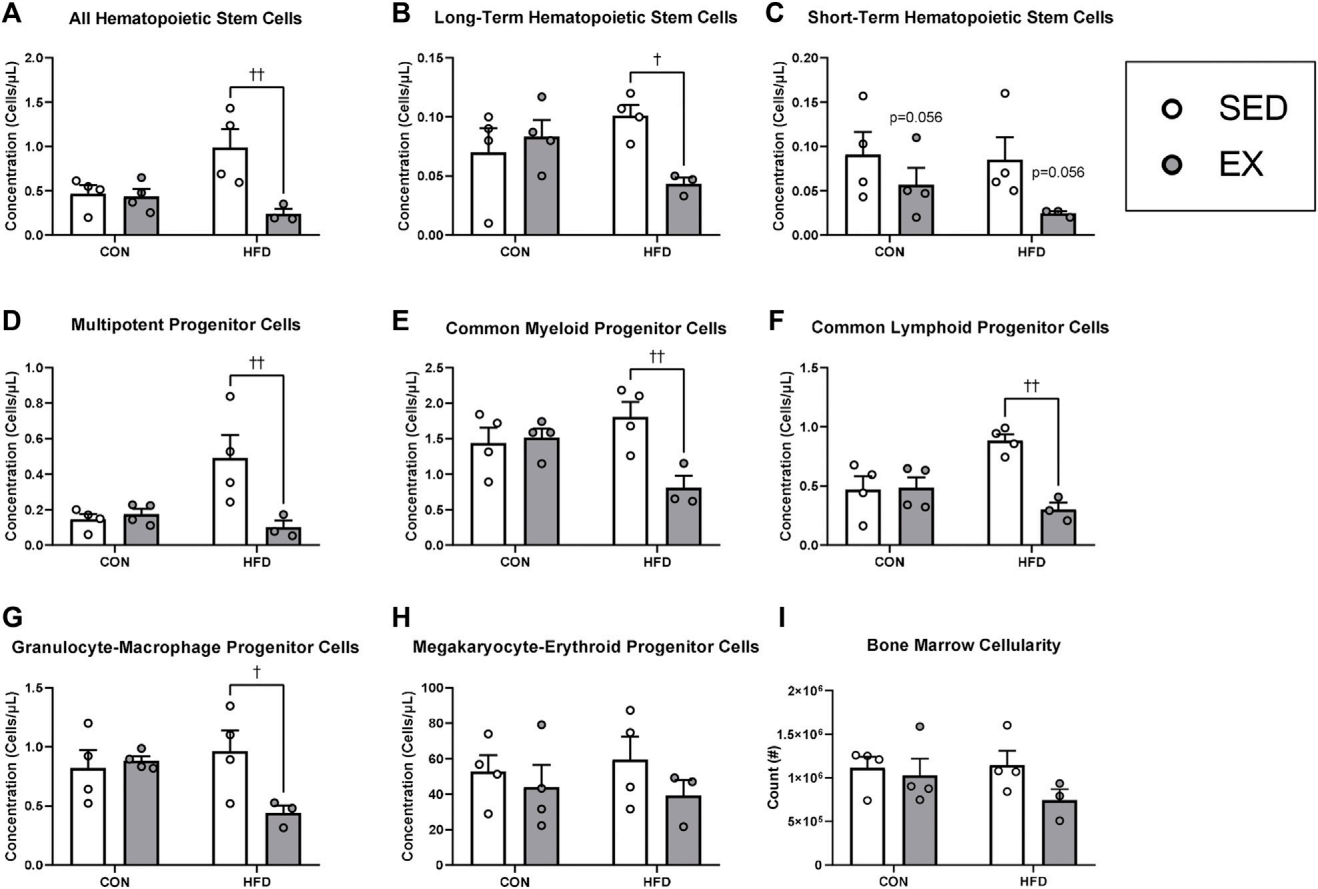
Extracellular vesicles from exercise-trained (EX) mice given a high-fat diet (HFD) return hematopoietic stem (HSC) and progenitor cell concentrations to control diet levels. **(A)** All HSCs, **(B)** long-term HSCs, **(C)** short-term HSCs, **(D)** multipotent progenitor cells, **(E)** common myeloid progenitor cells), **(F)** common lymphoid progenitor cells, **(G)** granulocyte-macrophage progenitor cells, **(H)** megakaryocyte-erythroid progenitor cells **(I)** total bone marrow cellularity. ^†^ *p* < 0.05 interaction effect; ^††^ *p* < 0.01 interaction effect (*n* = 4 per group, HFD-EX *n* = 3).

**FIGURE 4:**
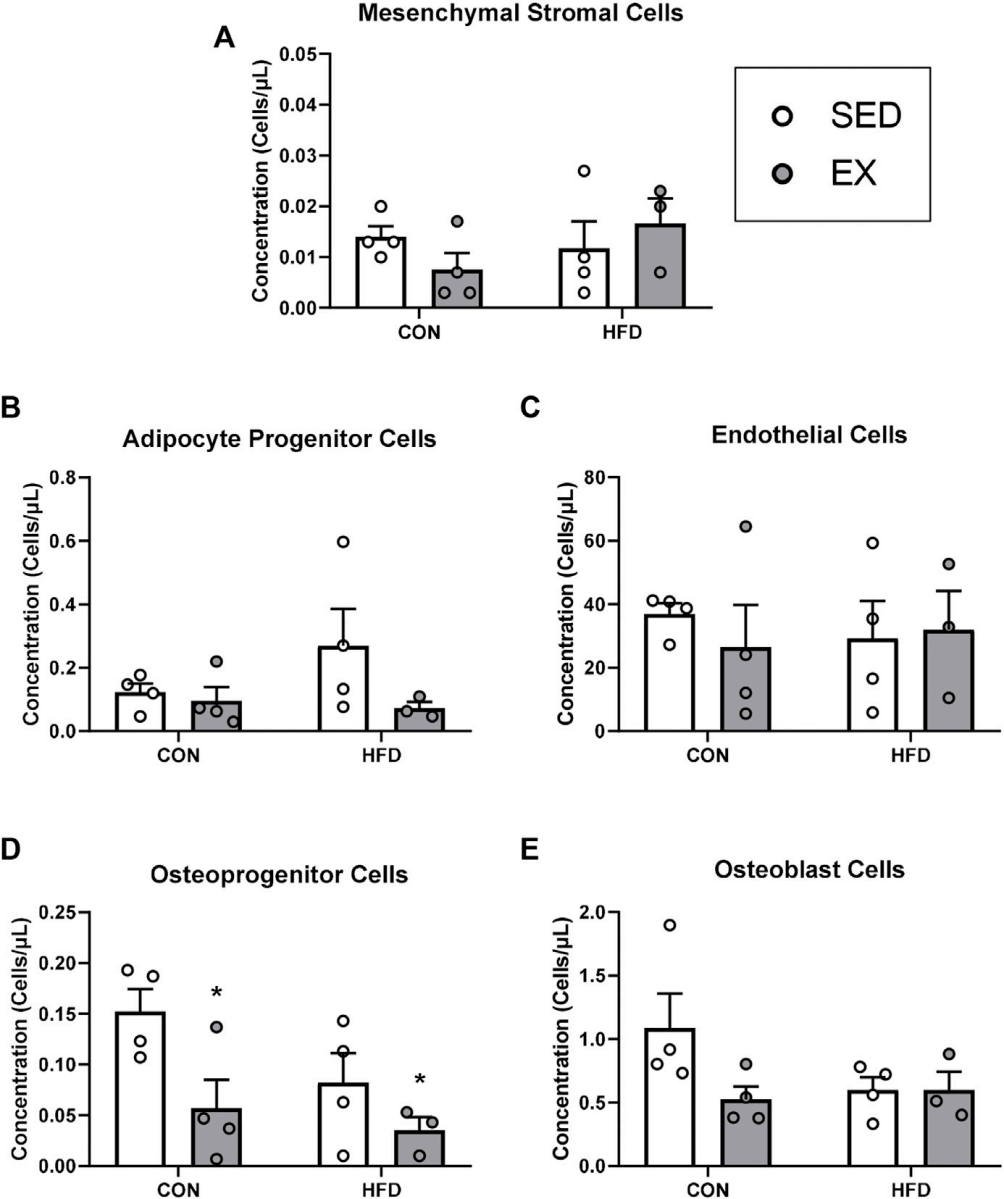
Extracellular vesicles from exercise-trained mice decrease osteoprogenitor cell concentrations. **(A)** Mesenchymal stromal cells, **(B)** adipocyte progenitor cells, **(C)** endothelial cells, **(D)** osteoprogenitor cells, and **(E)** osteoblast cells. *p < 0.05 main effect of exercise (*n* = 4 per group, HFD-EX *n* = 3).

Mice injected with HFD-EX EVs had lower concentrations of HSC (Figure 3A), long-term HSC (LT-HSC) (Figure 3B), multipotent progenitor (MPP) (Figure 3D), CMP (Figure 3E), common lymphoid progenitor (CLP) (Figure 3F), and granulocyte-macrophage progenitor cells (Figure 3G) compared to mice injected with HFD-SED EVs (all *p* < 0.05). There was also a trend for fewer short-term HSCs (ST-HSCs) following injection of EX EVs compared to SED (Figure 3C, *p* = 0.056). There were no effects of HFD or EX EVs on megakaryocyte-erythroid progenitor (MEP) cells (Figure 4H) or on total bone marrow cellularity (Figure 3I).

### Extracellular vesicles from exercise training mice reduce osteoprogenitor cell concentration during stress hematopoiesis

Mice injected with neither EX nor HFD EVs impacted MSC (Figure 4A), adipocyte progenitor (HFD-EX vs. HFD-SED interaction *p* = 0.14) (Figure 4B), endothelial (Figure 4C), osteoblast (Figure 4E) during stress hematopoiesis. Mice injected with EX EVs; however, had lower osteoprogenitor cell concentrations (*p* < 0.05) (Figure 4D).

### HFD Extracellular vesicles increase MSC adipogenic differentiation and EX EVs increase osteogenic differentiation

**FIGURE 5:**
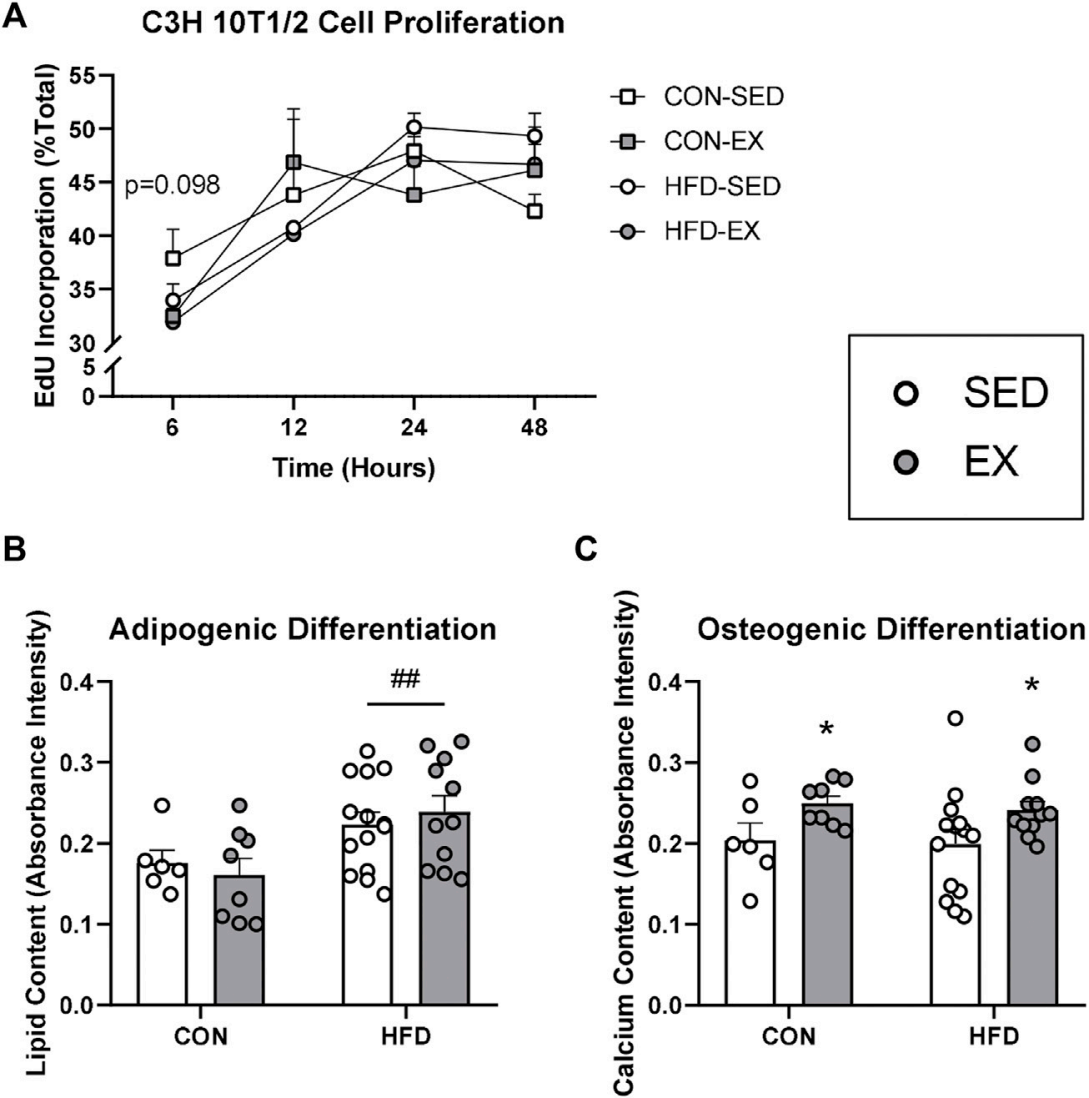
Extracellular vesicles (EVs) impact mesenchymal stromal cell (MSC) differentiation activity *in vitro.* C3H/10T1/2 MSCs were differentiated into either adipocytes and stained with Oil Red O or osteoblasts and stained with Alizarin Red S dye. **(A)** Proliferation by EdU stain at 6, 12, 24, and 48 h **(B)** EVs from high-fat diet-fed mice increased adipogenic differentiation and **(C)** EVs form exercise-trained mice increased osteogenic differentiation. *p < 0.05 main effect of exercise; ^##^ *p* < 0.01 main effect of high-fat diet (*n* = 6–14 per group).

Since EX EVs decreased osteoprogenitor cell concentrations, we investigated whether HFD and EX EVs directly impact MSC fate. There was no effect of HFD EVs or EX EVs on MSC proliferation, although a trend (*p* = 0.098) was seen for higher proliferation at 6 h post-Edu addition with CON-SED EVs compared to CON-EX EVs (Figure 5A). MSCs treated with HFD EVs *in vitro* had higher adipogenic differentiation (*p* < 0.01) compared to MSCs treated with CON EVs (Figure 5B). Conversely, MSCs treated with EX EVs had higher osteogenic differentiation (*p* < 0.05) compared to MSCs treated with SED EVs (Figure 5C).

### HFD-EX EVs have lower miR-193 and miR-331-5p content compared to HFD-SED Extracellular vesicles

**FIGURE 6:**
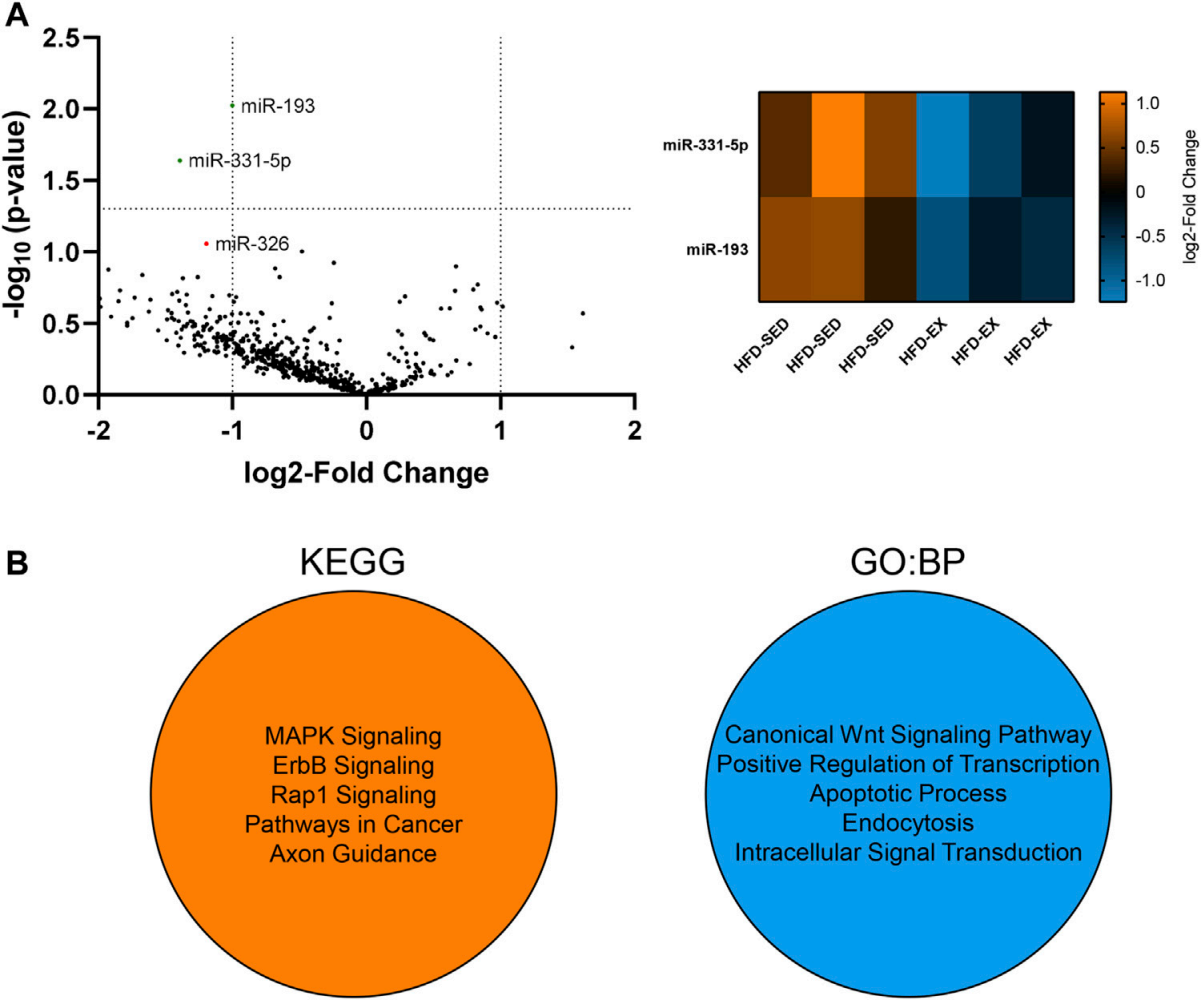
Extracellular vesicles (EVs) from high-fat diet exercise-trained (HFD-EX) mice have lower miR-331-5p (*p* < 0.05) and miR-193 (*p* < 0.01) expression compared to HFD-sedentary (HFD-SED) mice. **(A)** Volcano plot of all measured miRNAs and heatmap of differentially expressed miRNAs. miR-326 approached lower expression in HFD-EX EVs compared to HFD-SED (*p* = 0.0877). The vertical dotted lines represent the margin for physiologically meaningful expression changes as per default ROSALIND analysis. The horizontal dotted line represents statistical significance (*p* = 0.05). (*n* = 3 per group). **(B)** Kyoto Encyclopedia of Genes and Genomes (KEGG) and Gene Ontology: Biological Processes (BP) predicted target pathways by miRWalk combined analysis of miR-193 and miR-331-5p.

We conducted a high-throughput miRNA screen from EVs in the current study to determine if alterations in miRNA cargo may explain our observed alterations to the various bone marrow cell populations. We focused this screen on HFD-SED and HFD-EX based on changes observed in our flow cytometric results and to determine the extent to which exercise training could reverse the effects of HFD. Our analysis of 577 distinct mouse miRNAs revealed lower miR-193 (2.00 fold, *p* < 0.01) and miR-331-5p (2.63 fold, *p* < 0.05) content in HFD-EX (*n* = 3) compared to HFD-SED (*n* = 3) (Figure 6A).

Using miRWalk, we identified the top five predicted target pathways of both miR-193 and miR-331-5p combined by KEGG and GO:BP analysis (Figure 6B). KEGG analysis predicts miRNA control of genes and the affected general metabolic pathways while GO:BP predicts more detailed molecular functions. KEGG analysis showed predictive activity in the MAPK, ErbB, and Rap1 signaling pathways alongside pathways in cancer and axon guidance, while GO:BP analysis showed predictive interactions with the canonical Wnt signaling pathway, transcription regulation, apoptosis, endocytosis, and intracellular signal transduction.

## Discussion

Exercise and obesity induce significant alterations to bone marrow architecture and HSC activity (Styner et al., 2014; Emmons et al., 2016). Given that EVs regulate hematopoiesis (Wen et al., 2016; Szatmári et al., 2017), and exercise training increases circulating EV concentrations (Bei et al., 2017), we sought to understand the role of exercise- and high fat diet-induced EVs as molecular mediators of stress hematopoiesis and bone marrow remodeling. In diet-induced obesity, exercise-induced EVs reversed the effects of diet on HSC concentrations and induced MSC differentiation along the osteogenic lineage during stress hematopoiesis. Interestingly, HFD-induced obesity resulted in bone marrow EVs that promoted adipogenesis *in vitro*. We further identified down-regulation of miR-193 and miR-331-5p content in exercise-induced EVs from high fat diet-fed mice as a potential molecular target responsible for these effects. Together, our findings suggest that bone-marrow derived EVs from EX mice partially reverse the effects of HFD EVs on hematopoiesis and the HSC niche providing a novel mechanism for the effects of exercise on hematopoiesis.

Previous studies that have examined EVs in response to exercise have focused mostly on the effects of acute exercise-induce and circulating EVs on skeletal muscle adaptations to exercise (Nederveen et al., 2021). The present study provides the first description of exercise-induced EVs specifically from the bone marrow, and also the first investigation of the effects of exercise-induced EVs on hematopoiesis. Acute exercise studies have shown elevated circulating EV concentrations immediately following a stepwise cycling test to exhaustion that subsided at 90-min post-exercise (Frühbeis et al., 2015). However, similar to our findings in bone marrow, previous work has indicated that chronic exercise training does not result in any changes to total EV concentration but found an altered miRNA expression profile (Hou et al., 2019). As such, it appears that changes in total EV concentrations may be part of the normal stress response to unaccustomed exercise, while training may have a larger impact on EV cargo. Despite no alterations to total EV concentration with HFD or EX, we found a redistribution of EV size in response to EX with EX mice having higher EV concentration at 315 ± 15 and 345 ± 15 nm, and specifically in CON diet condition compared to HFD at 225 ± 15 nm. This size range corresponds to large EVs (>100 nm) which predominantly originate as microvesicles that are formed directly from plasma membrane budding (Ståhl et al., 2019). This origin disparity leads to varying cargo profiles (Bruschi et al., 2019) that differentially impact cell function (Wen et al., 2016). These data suggest EX alters EV release in a way that partially reverses the consequences of diet-induced obesity.

Previous reports from our lab and others indicate exercise impacts hematopoiesis *via* paracrine actions in the HSC niche (Emmons et al., 2016; Wen et al., 2016; Szatmári et al., 2017; Frodermann et al., 2019). Therefore, to determine the role of EVs in these effects, we injected the isolated EVs from HFD and EX mice into naïve mice undergoing radiation-induced stress hematopoiesis. We injected EVs 24 h post-irradiation because Wen and colleagues had previously shown that injecting EVs at this timepoint following radiation altered hematopoiesis (Wen et al., 2016). Similar to previous work examining the effects of exercise in obesity on hematopoiesis (Frodermann et al., 2019), we found that exercise-induced EVs attenuated hematopoiesis in the HFD condition as evidenced by reduced concentrations of total HSC, LT-HSC, MPP, and CLP concentrations. Previous work from our lab used a similar model to the current study, where mice instead were given 3 Gy irradiation after 8 weeks of HFD or CON diet interventions and 4 weeks of EX or SED, while continuing the EX and dietary interventions for another 4 weeks after radiation (Emmons et al., 2019). Interestingly, in that study we found that EX increased LT-HSC, ST-HSC, MPP, and CLP cell counts without an effect of HFD (Emmons et al., 2019). Discrepancies in these studies could be due to different durations of exercise training, the direct effects of exercise *versus* indirect effects of exercise-induced EVs, or the age when the mice were irradiated. In the current study mice were irradiated at 5 weeks of age while the mice in the previous study were irradiated at 13 weeks of age. These ages represent mice pre- and post-sexual maturity (Dutta and Sengupta, 2016), which may impart differing hematopoietic and MSC differentiation responses to irradiation, exercise, and high-fat diets.

Our lab and others have previously determined that the effects of exercise on hematopoiesis are mediated by alterations in cellular communication in the bone marrow (Yamazaki et al., 2011; Kunisaki et al., 2013; Bruns et al., 2014; Hérault et al., 2017; Emmons et al., 2019). As a first step towards understanding if EVs were influencing hematopoiesis indirectly through HSC niche cells, we quantified MSCs, their progeny (i.e., osteoprogenitor, adipocyte progenitor, and osteoblast cells), and endothelial cells. These analyses revealed that EX EVs decreased osteoprogenitor cell concentrations with no effects of EX- or HFD-induced EVs on other bone marrow cell populations. HFD is known to increase bone marrow adipose tissue content which can be prevented (Styner et al., 2014; Emmons et al., 2019) or reversed (Emmons et al., 2018) by exercise training. Conversely, exercise training is known to increase osteogenesis from MSCs (Baker et al., 2011; Zhang et al., 2020), thus we hypothesized that the lower concentration of osteoprogenitors in the mice that received exercise-induced EVs may be due to enhanced differentiation. Our *in vitro* findings support this hypothesis showing that treating MSCs with exercise-induced EVs enhanced their osteogenic differentiation. Previous work has suggested that enhanced osteogenic differentiation in response to exercise training is due to mechanotransduction signaling in MSCs (Sen et al., 2011), thus our findings provide a novel paracrine mechanism whereby exercise may be enhancing osteogenesis. Interestingly, our *in vitro* analyses also revealed a role for HFD-induced EVs in enhancing adipogenesis which provides an additional mechanism explaining the enhanced adipogenic differentiation of MSCs and accumulation of marrow adipose tissue in HFD-induced obesity (Emmons et al., 2019).

As previous studies have shown alterations to cell function through variations to EV cargo (Hou et al., 2019), we reasoned that the exercise-induced effects were partially due to alterations in EV cargo. Previous studies have examined muscle-derived EV cargo in the context of exercise, yet the EV cargo of the bone marrow environment remains unexplored. Here, we examined the miRNA cargo of the isolated EVs due to their established role in cell signaling (Avraham and Yarden, 2012; Vu et al., 2019). We found miR-331-5p (*p* < 0.05) and miR-193 (*p* < 0.01) downregulation in HFD-EX EVs compared to HFD-SED EVs. Correlational findings from clinical studies suggest lower miR-331-5p expression is related to leukemia relapse (Feng et al., 2011) and worsened responses to therapies for acute myeloid leukemia (Butrym et al., 2015). Previous data from our lab has shown that acute myeloid leukemia incidence is increased with HFD, that is, attenuated by exercise throughout the lifespan (Farber et al., 2021), thus downregulated miR-331-5p expression in HFD-EX EVs may be implicated in this observation. Relatively more information is available regarding miR-193 and hematopoiesis. Haetscher and colleagues found that miR-193b is upregulated in mouse hematopoietic stem cells while miR-193a expression is found primarily in committed myeloid cells, such as monocytes and granulocytes (Haetscher et al., 2015). Along these lines, overexpression of miR193a in HSCs impaired their regenerative capacity in transplantation assays, but had augmented granulocytic differentiation *in vitro* (Krowiorz et al., 2018), suggesting mirR-193b may be involved in maintaining HSC stemness, while mir-193a may be involved in myeloid differentiation.

miR-193a also plays a role in regulating MSC fate by suppressing osteogenic differentiation in human MSCs (Wang S.-N. et al., 2018). Further, miR-193a downregulation induced osteoblast differentiation (Wang W. et al., 2018). These findings align nicely with our data as miR-193 was lower in exercise-induced EVs, and exercise-induced EVs promoted osteogenesis. miR-193a-3p has been shown to downregulate leucine-rich repeat-containing G-protein coupled receptor 4 (LGR4) and activating transcription factor 4 (ATF4) (Wang W. et al., 2018). LGR4 nonsense mutations have previously been implicated decreased bone mineral density and elevated risk of osteoporotic fractures (Styrkarsdottir et al., 2013), and ATF4 has been reported to positively regulate HSC expansion in mouse fetal liver (Zhao et al., 2015). miR-193a-3p downregulation may be promoting *lgr4* and *atf4* gene transcription to increase osteogenesis as we observed *in vitro*. Our KEGG analysis predicted the MAPK signaling pathway as a potential target for miR-331-5p and miR-193 which is corroborated by work from Lv and colleagues showing that miR-193a-3p downregulation increases MAPK signaling (Lv et al., 2020). The MAPK signaling cascade is important for cell proliferation and differentiation. The pathway is composed of multiple components, including JNK and ERK. Previous reports showed elevated JNK signaling promotes early MSC osteogenic differentiation (Brito et al., 2019; Mizerska-Kowalska et al., 2019), and others have shown that ERK inactivation in osteoprogenitors leads to decreased bone mass (Kim et al., 2019). miR-193 and 331-5p downregulation may be partially responsible for the upregulated osteogenesis seen *in vitro* in the current study.

In conclusion, our results indicate that exercise training-induced EVs restore normal hematopoiesis and enhance osteogenic differentiation in the context of HFD-induced obesity. Mechanistically, these findings may be explained by elevated large EV release alongside downregulated miR-193 and miR-331-5p content in HFD-EX EVs compared to HFD-SED EVs. These results provide a novel mechanism the regulation of hematopoiesis and MSC fate by exercise. Future studies should identify the cellular source of these exercise-induced EVs to better-understand how intercellular crosstalk occurs between HSCs and their niche cells in the context of exercise training.

## Data Availability

The raw data supporting the conclusions of this article will be made available by the authors, without undue reservation.
