# Supplementary figures and images for: The role of exercise-and high fat diet-induced bone marrow extracellular vesicles in stress hematopoiesis
Source: Front Physiol. 2022 Nov 23;13:1054463. doi: 10.3389/fphys.2022.1054463 (PMC9728614; doi:10.3389/fphys.2022.1054463)

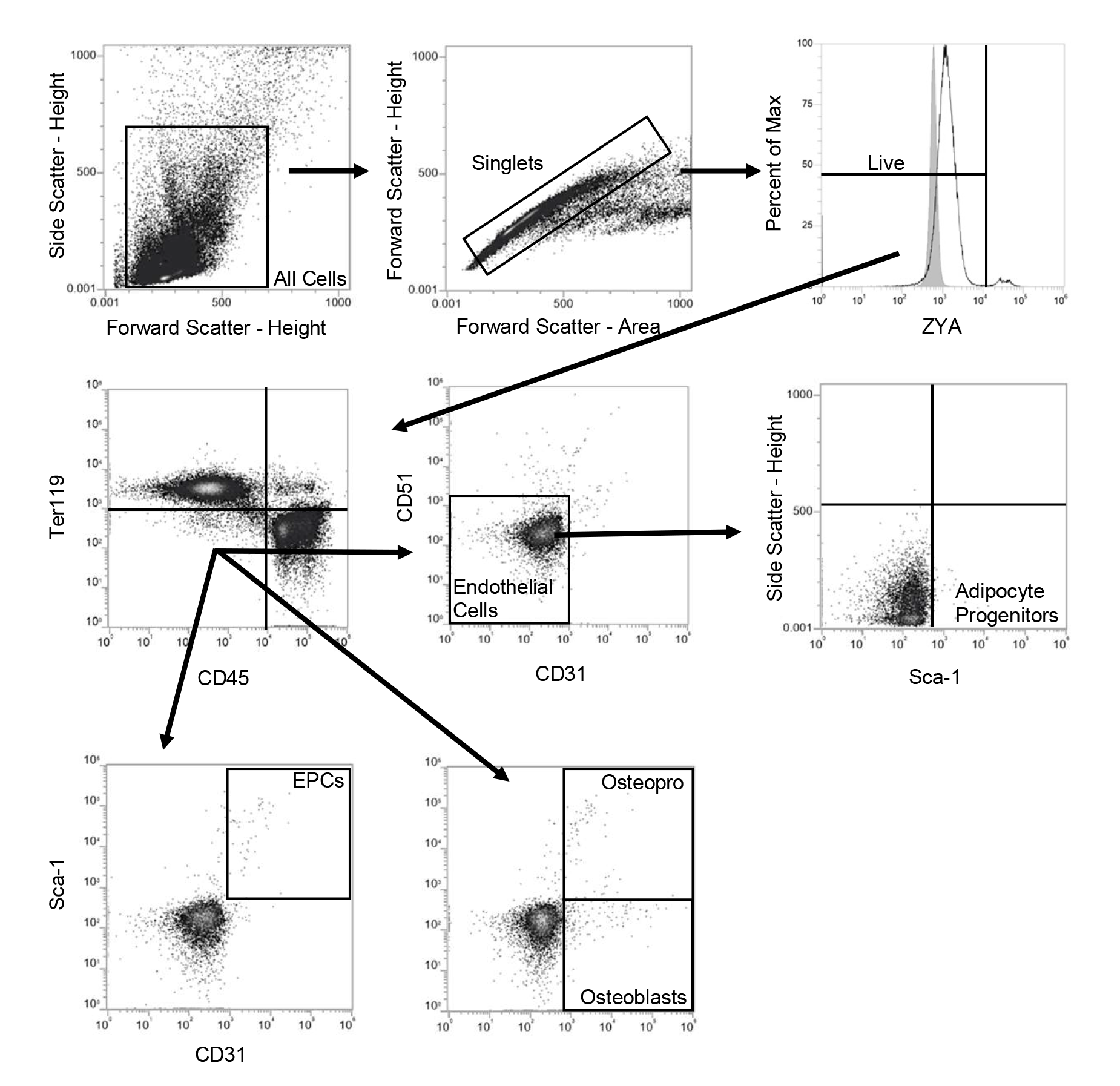

Supplement: Supplementary file 1 [file Image3.TIF]

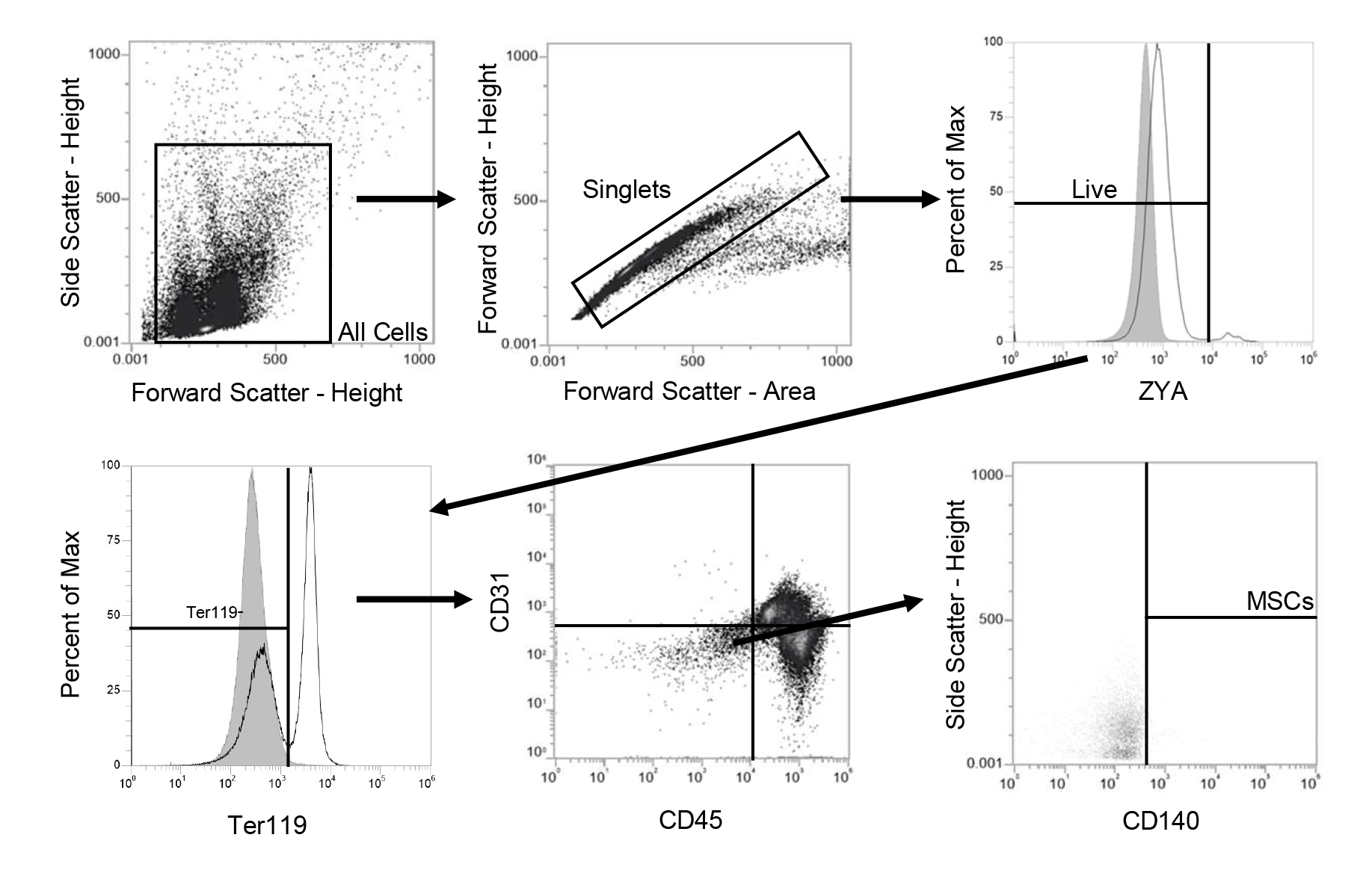

Supplement: Supplementary file 2 [file Image2.TIF]

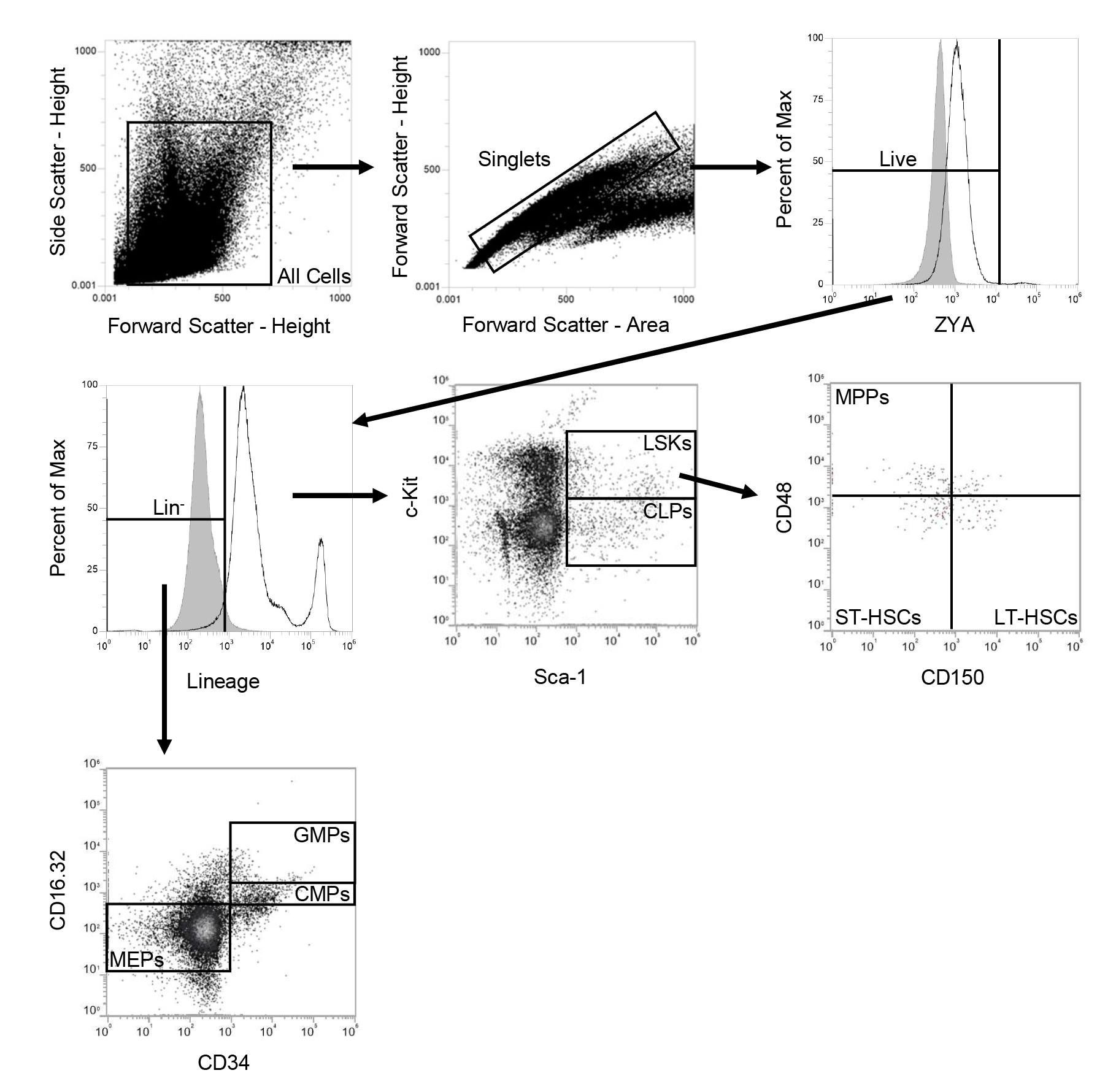

Supplement: Supplementary file 3 [file Image1.TIF]
